# CO_2_ responsive materials in oilfield engineering: synthesis, mechanisms, and applications

**DOI:** 10.1039/d5ra03359d

**Published:** 2025-06-30

**Authors:** Qiang Li, Xuanze Zhu, Jiuyi Chen, Xionghu Zhao

**Affiliations:** a Petroleum College, China University of Petroleum-Beijing at Karamay Karamay 834000 China

## Abstract

The increasing demand for oil and gas resources, coupled with growing concerns over the environmental impact of conventional chemical agents, has heightened the need for sustainable alternatives. CO_2_ responsive materials, which utilize CO_2_ as an environmentally friendly stimulus, have emerged as promising solutions for improving chemical performance while minimizing environmental impact in petroleum engineering. This review systematically examines the functional groups, response mechanisms, and synthesis strategies of CO_2_ responsive polymers in oil and gas operations, with particular emphasis on their applications in drilling and reservoir engineering. The review explores the relationship between the reversibility of CO_2_ responsive materials and their environmental adaptability, focusing on applications in cementing, oil–water separation, gas channeling plugging, viscosity modification, and enhanced oil recovery. By evaluating response mechanisms and environmental adaptability, this work offers valuable insights into the optimization of CO_2_ responsive materials for practical use in petroleum operations. Additionally, challenges such as response sensitivity and long-term stability are critically explored, and potential solutions and strategies are proposed. The findings aim to support the low-carbon transformation of the oil industry and promote the adoption of sustainable practices in hydrocarbon extraction.

## Abstract 2

This review focuses on CO_2_ responsive materials in petroleum engineering, discussing their synthesis, mechanisms, and applications to promote sustainable oil extraction and low-carbon transformation.

## Graphical abstract

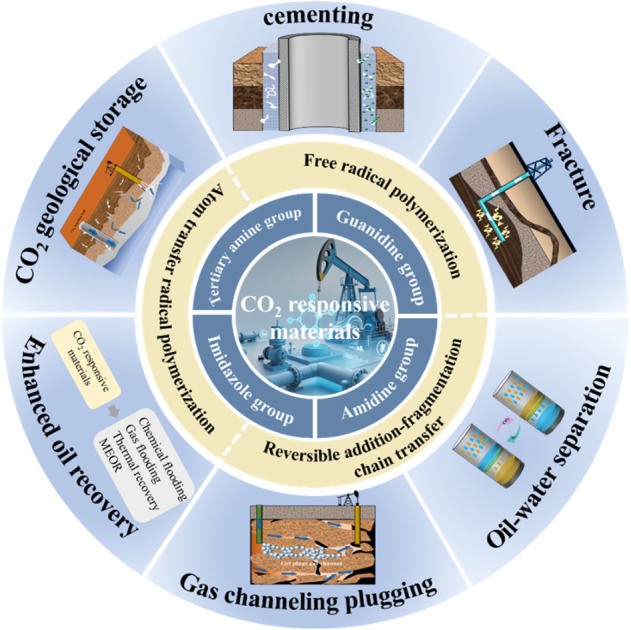

## 1 Introduction

Energy is the lifeblood of modern society, supporting both global economic growth and social progress.^1^ Global energy consumption has surged from less than 4 billion tons of oil equivalent in 1965 to nearly 14 billion tons in 2018.^2^ Fossil energy has consistently dominated the global energy mix, accounting for over 83% of total energy consumption, with oil and gas resources making up more than 50% of this share.^3^ Studies have shown that human dependence on fossil energy sources will continue to increase over the next 20 years, with demand for oil and natural gas expected to grow at an average annual rate of 0.7% and 1.2%, respectively.^4,5^ The growth in oil and gas consumption is significantly positively correlated with crude oil extraction, however, the extraction process itself contributes 15–40% of global greenhouse gas emissions, primarily in the form of CO_2_.^6^ With the continuous accumulation of greenhouse gases, a series of major problems have arisen, including sea level rising, ocean acidification, and global warming. Climate and environmental issues are related to the common destiny of mankind.^7–9^ In response to these challenges, CO_2_ responsive materials have emerged, driven by efforts to improve energy efficiency and promote renewable energy development. These materials offer the potential to transform CO_2_ from an environmental burden into an engineering advantage and to facilitate the transition to smarter and more sustainable oil and gas development technologies.^10,11^

CO_2_ responsive materials are a class of smart materials that produce reversible responses to changes in CO_2_ concentration. These materials achieve this functionality by incorporating CO_2_ sensitive groups into polymer chains.^12–14^ CO_2_ responsive materials utilize CO_2_ as a stimulating factor, eliminating the need for exogenous chemical additives. They primarily combine multiple materials through protonation/deprotonation mechanisms to achieve variable performance regulation.^15,16^ In the 2010s, CO_2_ responsive materials began to be applied to wellbore sealing and fluid viscosity enhancement on a small scale.^17,18^ As global energy demand rises and conventional shallow fossil resources are gradually depleted, oil exploration is expanding into complex reservoirs, such as low-permeability and ultra-deep formations. Consequently, the application scope of CO_2_ responsive materials is also broadening.^19^ The latest applications of CO_2_ responsive materials in oil and gas fields are mainly concentrated in drilling, cementing, oil production, enhanced oil recovery and oil–water separation. In the cementing stage, CO_2_ responsive materials can be triggered to repair cement microcracks through a self-repairing response. These materials are also applied in Carbon Capture, Utilization, and Storage (CCUS) technology to seal leaking layer;^20–22^ during the fracturing stage, the viscosity of the fracturing fluid in the formation can be enhanced by combining CO_2_ responsive materials with surfactants and foams. Once the proppant migrates to the designated location, the fluid viscosity can be rapidly reduced by injecting N_2_, thus minimizing reservoir damage.^23,24^ For emulsions produced during oil and gas extraction, CO_2_ responsive materials can trigger the hydrophilic/hydrophobic dynamic switching of the emulsions to disrupt the emulsification interface and promote oil droplets aggregation, thus enhancing the efficiency of the oil–water separation.^25^

**Fig. 1:**
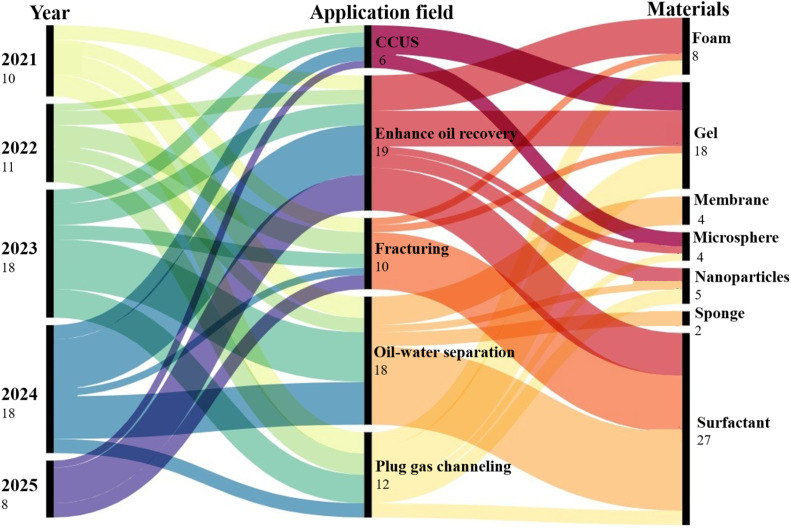
Publications related to CO_2_ responsive materials in petroleum extraction (2021–2025).

Recent research on the application of CO_2_ responsive materials in petroleum industry has shown significant growth, particularly in drilling and reservoir engineering. Publications from the Google Scholar database are analyzed to assess the current research status of CO_2_ responsive materials, with the statistical period spanning from 2021 to 2025. A customized query was employed to search for relevant articles, incorporating keywords such as CO_2_ responsive materials, drilling fluids, fracturing fluids, oil–water separation, plugging profile control, enhanced oil recovery, and CCUS. A total of 65 valid documents were retrieved (Fig. 1). The findings reveal that the primary application areas of CO_2_ responsive materials in oilfield are enhanced oil recovery, fracturing, and sealing, with increasing research focus on CCUS and oil–water separation in recent years. The main types of CO_2_ responsive materials that have been utilized include surfactants, gels, foams, and nanoparticles.

The gradual application of environmentally responsive smart materials in the oil field provides new ideas for solving complex formation challenges. In recent years, CO_2_ responsive materials have become a research hotspot in the field of oilfield smart materials due to their environmental friendliness and controllability. H. Liu *et al.*^13^ systematically sorted out the chemical properties of CO_2_ responsive polymers, conducted in-depth analysis from the response mechanism at the molecular level to the synthesis path, and explored the potential application direction of CO_2_ responsive gels based on their self-assembly characteristics. Yang *et al.*^26^ focused on the application progress of CO_2_ responsive materials in the separation field, and innovatively proposed the feasibility of their synergistic effect with surfactants in oil–water separation and unconventional oil and gas development, providing an important reference for functional design. Jansen-van Vuuren *et al.*^27^ comprehensively summarizes the preparation, properties and applications of CO_2_ responsive gels. However, existing reviews have yet to fully address the emerging applications of CO_2_ responsive materials in oil and gas extraction, while their engineering adaptability under complex reservoir conditions remains to be systematically integrated.^28^ The value and novelty of this review lies in its key differences from existing reviews on this topic: (1) a brief description of the existing CO_2_ responsive polymer action mechanisms and synthesis methods that are widely used in petroleum engineering; (2) the progress in the application of gels, surfactants, membranes, and nanoparticles related to CO_2_ response in drilling engineering, reservoir engineering, and produced fluid treatment; (3) a comprehensive categorization and outlook for the last five years of the specific applications of CO_2_ responsive materials in the field of oil and gas, which provides support for the green transformation of the oil and gas industry.

**Fig. 2:**
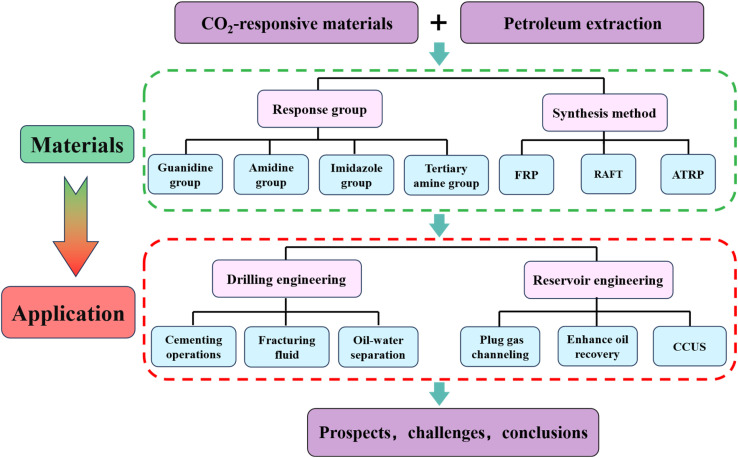
The logical structure of this review work.

The purpose of this review is to systematically sort out the development of CO_2_ responsive materials in oil and gas engineering over the past five years, focusing on the suitability of their response mechanisms for the oilfield environment, and their engineering contributions to extreme reservoirs and low-carbon targets. This paper emphasizes the material systems such as gels, foams, and membranes prepared based on CO_2_ responsive functional groups, provides an overview of the current status and challenges of their field applications in oilfields. In this work, the first part after the introduction summarizes in detail the common CO_2_ sensitive groups and synthesis methods for CO_2_ responsive polymers. The second section reviews the current status of CO_2_ responsive materials in drilling and reservoir engineering. Finally, the prospects and challenges of CO_2_ responsive materials in the oil and gas field are discussed. Fig. 2 illustrates the structure of this review.

## 2 CO_2_ responsive polymer synthesis

### 2.1 CO_2_ responsive groups

**Fig. 3:**
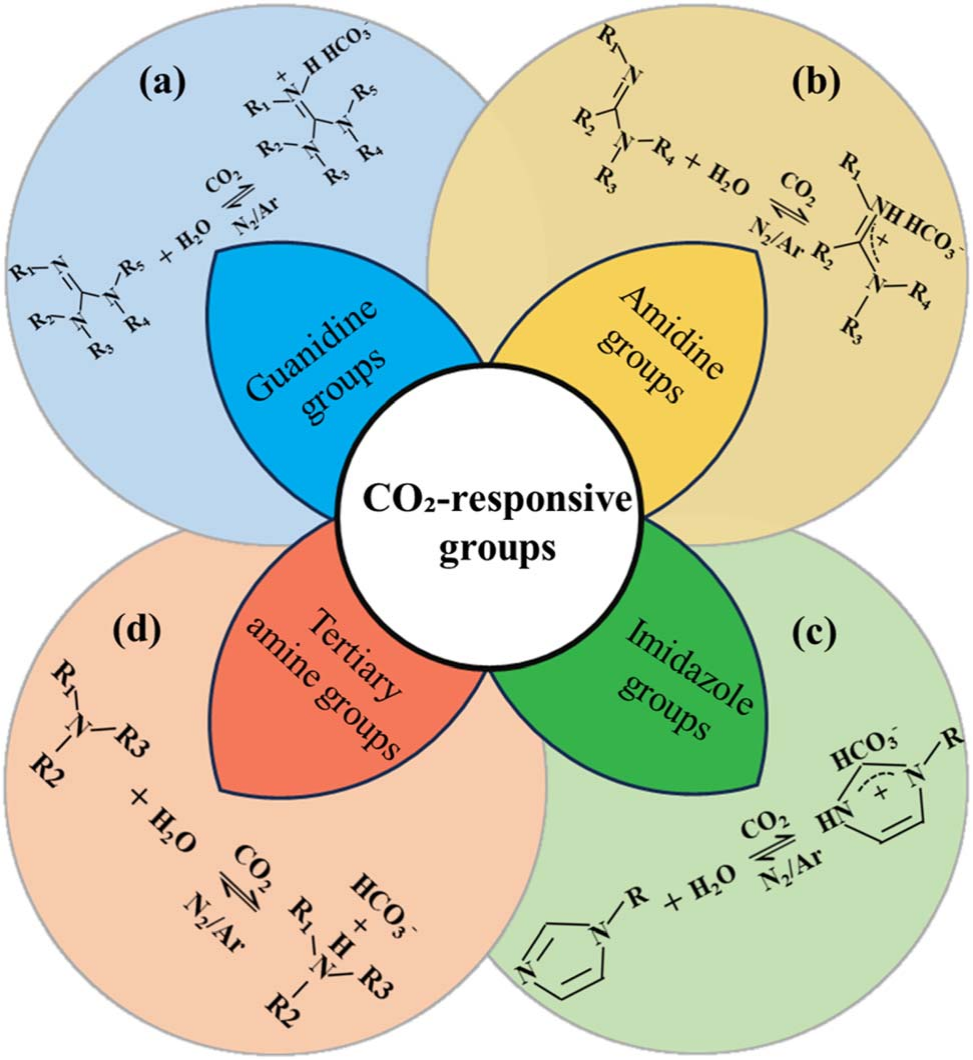
Reversible response mechanism of CO_2_ responsive functional groups.

CO_2_ responsive functional groups play a crucial role in the design of smart polymeric materials, as they facilitate dynamic and reversible interactions with carbon dioxide. These interactions enable precise control over material properties in response to various environmental stimuli, thereby enhancing CO_2_ sensitivity and intelligent functionality. Traditionally, the specific response mechanism can be described as Brønsted acid-base theory.^29^ Based on the nature of their CO_2_ responsive functional groups, CO_2_ responsive polymers can be classified into four types: guanidine group, amidine group, imidazole group and tertiary amine group (Fig. 3).

#### 2.1.1 Guanidine group

Guanidinium-based CO_2_ responsive polymers regulate hydrophilicity through the reversible protonation reaction of the guanidinium group (R–NH–C(=NH)–NH_2_) in the presence of CO_2_ : when CO_2_ dissolves in water, CO_2_ hydrates to form H^+^/HCO_3_^−^ (CO_2_ + H_2_O ⇌ H^+^ + HCO_3_^−^), the guanidinium group protonates to form R–NH–C(=NH)–NH_3_^+^, which drives hydrophilic swelling of the polymer. Conversely, when subjected to N_2_ purging and heating, the guanidinium group deprotonates to restore hydrophobic contraction (Fig. 3a).^30^ This mechanism can be applied in petroleum engineering to realize intelligent control of fluid flow.^31,32^ CO_2_ injection induces protonation-driven viscosity surge or gelation to block high-permeability channels, redirecting fluids to low-permeability zones; CO_2_ withdrawal restores low-viscosity states, minimizing reservoir damage.^33,34^ Reacting cyanamide (NH_2_CN) with amine-functionalized supports (*e.g.*, aminated silica/carbon) in toluene/xylene at 110–130 °C for 6–12h under N_2_ is a recommended industrial synthesis method. Catalysts like AlCl_3_ accelerate imine intermediate formation, yielding surface-grafted guanidine. These materials exhibit superior performance in high- temperature and high salinity reservoirs, however, their pH sensitivity and synthetic complexity require further optimization to broaden their applications.

#### 2.1.2 Amidine group

The amidine moiety (–NH–C(=NH)–NH_2_, Fig. 3b), a strongly basic nitrogen-rich functional unit, exhibits highly efficient CO_2_ responsive behavior due to its planar conjugated structure and reversible protonation. In aqueous media, CO_2_ hydrates to form H^+^ and HCO_3_^−^. The amidine group forms a protonated cation (–NH–C(=NH)–NH_3_^+^) by trapping the H^+^, which significantly enhances the hydrophilicity and induces the polymer to solubilize. After removal of the CO_2_ (*e.g.*, *via* N_2_ blowing or heating), the deprotonation restores the hydrophobic state, driving material shrinkage or precipitation. The response is efficient and reversible under mild conditions (pH = 7–9, room temperature), with fast kinetics and broad pH adaptability, making the amidine-based polymers ideal for smart materials applications. The CO_2_ responsive behavior of amidine-based polymers was first reported by Y. Liu *et al.*^35^ Subsequent studies have refined their responsive design through atom transfer radical polymerization (ATRP) of functional monomers, such as *N*-amidinododecylacrylamide.^36^ However, the synthetic complexity and hydrolysis sensitivity of amidine-based polymers limit their scale-up applications--nitrile substrates (*e.g.*, acrylonitrile copolymers) react with anhydrous HCl in ethanol at 0–5 °C, followed by amine addition at 25 °C for 4 h, where yields amidinium salts hydrolyzed to free amidine using NaOH. In order to improve the stability of amidine polymers, it is necessary to optimize their chemical structure through molecular design, such as incorporating rigid backbones or protective groups, to expand their potential in engineering applications, including CO_2_ flooding and oil–water separation.^37,38^

#### 2.1.3. Imidazole group

The imidazole group, a five-membered heterocyclic structure with dual nitrogen atoms at 1,3-positions (Fig. 3c), exhibits dynamic CO_2_ responsiveness *via* protonation at the N-3 site. When CO_2_ dissolves in water, it forms H_2_CO_3_, which dissociates to release H^+^ that protonates imidazole, converting it into a positively charged imidazolium species (–NH^+^–CH–N–). This protonation significantly enhances hydrophilicity and induces material phase transitions, such as gel swelling or micelle dissociation. Subsequent CO_2_ removal triggers deprotonation, restoring the hydrophobic state for reversible modulation. Compared to amidine and tertiary amine groups, imidazole's higher basicity (p*K*a ∼7) facilitates CO_2_ response under mild pH conditions, with greater stability of protonated product.^39^ Reversible addition–fragmentation chain transfer (RAFT) polymerization enables the incorporation of imidazole groups into polymer chains, such as histamine-modified side-chain polymers, where CO_2_-activated protonated salts drive macroscopic dissolution or assembly transitions.^40^ Debus–Radziszewski Reaction–Condense glyoxal (40%), formaldehyde (37%), and ammonia/alkylamines in aqueous acetic acid at 70–80 °C for 5 h, where products (*e.g.*, 1-butylimidazole) are purified *via* vacuum distillation, is widely used to industrially synthesize it. The biocompatibility of imidazole groups has been utilized in smart drug delivery systems. Their application in petroleum engineering holds significant potential for reducing pollution and enhancing environmental protection.

#### 2.1.4 Tertiary amine group

Tertiary amine group (Fig. 3d) undergoes reversible protonation due to its weak basicity. The tertiary amine (R_3_N) captures H^+^ to form a protonated quaternary ammonium salts (R_3_NH^+^), which binds with HCO_3_^−^ to generate bicarbonate complex (R_3_NH^+^ HCO_3_^−^). Upon CO_2_ removal, deprotonation of the quaternary ammonium salt restores the hydrophobic state. Compared with primary and secondary amines, tertiary amines have lower basicity and can achieve reversible conversion between protonation and deprotonation at low temperature. In contrast, primary amines rely on carbamate salt bridges for gelation, requiring heating or inert gas purging for recovery.^41^ The tertiary amine group are produced using well-established synthetic techniques–reacting primary/secondary amines (*e.g.*, octylamine) with formaldehyde and formic acid at 100 °C for 6h and tertiary amines are extracted with diethyl ether and dried over MgSO_4_, and offer several advantages in terms of availability, environmental compatibility, and synergistic interactions with anionic surfactants. These interactions can lead to the formation of worm-like micelles or three-dimensional networks, thereby enhancing their applicability.^42,43^ However, several challenges such as high concentration requirements, long-term stability, and adaptability to complex reservoirs require further optimization through hydrophobic chain incorporation or functional copolymer design. Rahmatabadi *et al.*^44^ used polyethylene glycol (PEG) grafted onto carbon nanotubes (CNTs) to enhance the thermomechanical properties of composites. This strategy can also be applied to modified tertiary amine groups, because hydrophilic PEG segments can reduce the aggregation of tertiary amine-containing polymers in aqueous solutions, thereby solving the challenges of high concentration requirements mentioned above. Simultaneously, the steric hindrance of the PEG chain can not only be used to inhibit intermolecular hydrophobic interactions, but also regulate the protonation kinetics of the tertiary amine group, optimizing its responsiveness at different CO_2_ concentrations.

In conclusion, tertiary amine group is predominant in the field of intelligent drive modulation, particularly for CO_2_-triggered viscosity enhancement, gelation plugging and plugging profile control due to its low cost, ease of synthesis and reversible response at room temperature. The guanidine and amidine groups offer distinct advantages in high-salt reservoir plugging and high-temperature micellar viscosification respectively, but are limited by energy consumption and stability. The imidazole group has been expanded for applications in high-temperature CO_2_ capture and environmental protection treatment through ionic liquid design. Based on the characteristics of these response groups, a variety of highly efficient, environmentally adaptable, and multifunctional CO_2_ responsive technologies have been developed and applied in engineering. Table 1 presents a comparison of the advantages and limitations of four CO_2_ responsive groups.

**Table 1:** Comparative analysis of CO_2_ responsive groups for oilfield applications

Group type	Response mechanism	Advantages	Limitations	Primary applications	Ref.
Guanidine	CO_2_-triggered protonation enhances hydrophilicity; reversible hydrophobic recovery under high temperature	High thermal and salt tolerance; rapid response	Requires heating for reversal; complex synthesis; hydrolysis risk	High-temperature gas channeling plugging; CO_2_ selective membranes	30 and 45
Amidine	Reversible protonation/deprotonation at ambient conditions	Fast kinetics; broad pH compatibility; synergized effects with surfactants	Limited adaptability in alkaline environments; long-term stability	High-temperature micellar viscosity enhancement; smart foam flooding	35 and 46
Imidazole	Protonation *via* nitrogen lone pairs enables hydrophilic/hydrophobic switching	Biocompatibility; multifunctional design potential; high-temperature resistance	pH sensitivity; complex synthesis; high cost	High-temperature CO_2_ capture; ionic liquid-based oil displacement	39, 40 and 47
Tertiary amine	CO_2_-induced quaternary ammonium salt formation and dissociation	Low-cost synthesis; ambient reversibility; environmental adaptability	High concentration; performance degradation in complex reservoirs	Plugging profile control; viscoelastic micelle systems; mobility control	42 and 48

### 2.2 Synthesis method of CO_2_ responsive polymer

**Fig. 4:**
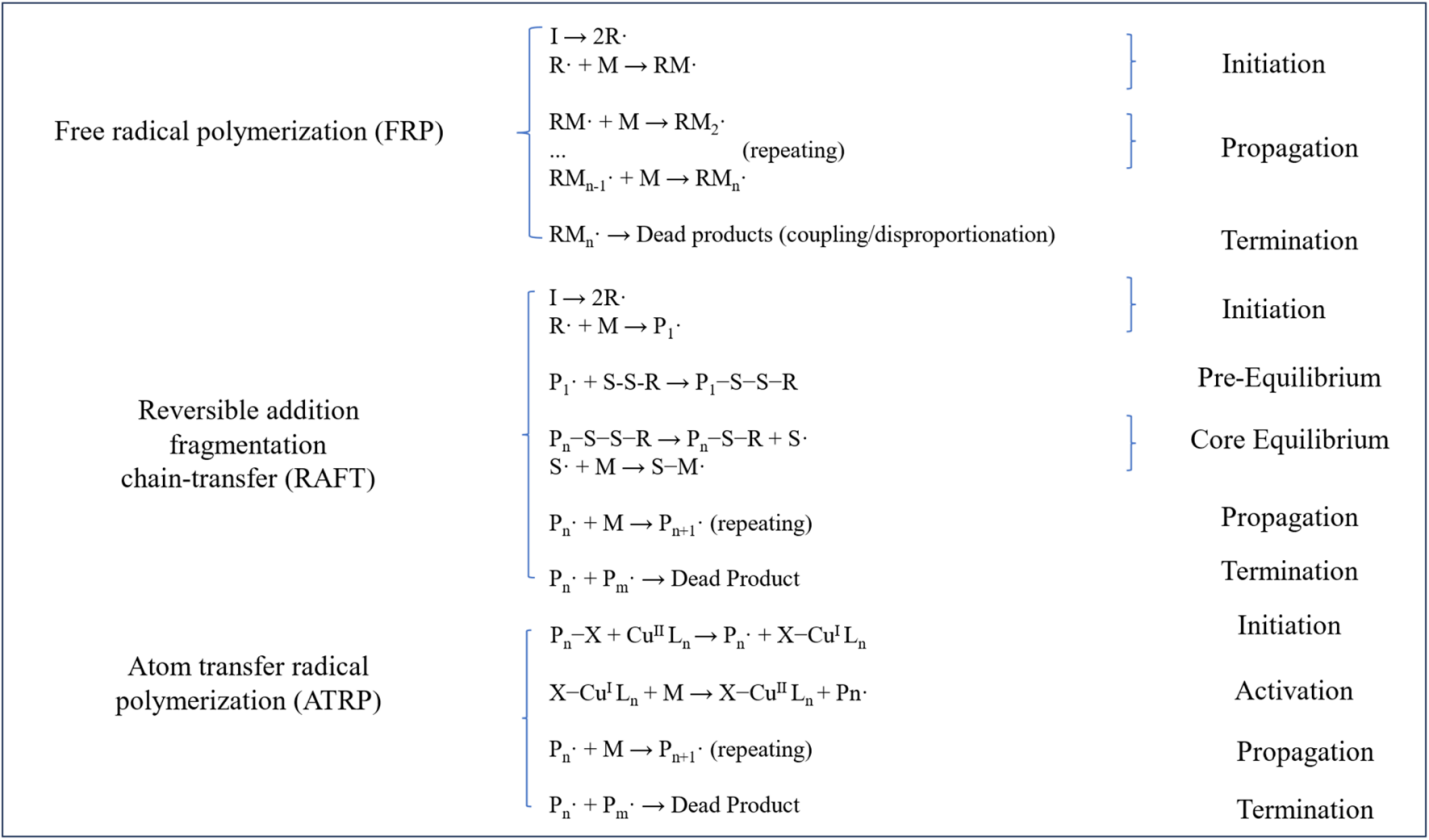
Synthesis of CO_2_ responsive materials.

The synthesis of CO_2_ responsive polymers focuses on incorporating functional groups into polymer structure to achieve reversible interactions with CO_2_. Several researchers have classified synthesis approaches into two categories: pre-modification and post-modification methods.^49,50^ Pre-modification methods consist of various polymerization techniques, including free radical polymerization (FRP), reversible addition–fragmentation chain transfer (RAFT), atom transfer radical polymerization (ATRP), and nitroxide-mediated radical polymerization (NMP).^13,51^ In contrast, post-modification methods primarily rely on post polymerization modification techniques, such as click chemistry and other related reactions to introduce CO_2_ responsive groups.^52^Fig. 4 demonstrates the possible reaction chains in terms of main synthesis methods, illustrating the basic industrially practical synthesis chains and fundamental parts of responsive groups including FRP, RAFT, and ATRP.

#### 2.2.1 Free radical polymerization

Free radical polymerization (FRP) is a widely utilized chain-growth polymerization technique that relies on the generation of reactive free radicals to initiate and propagate polymer chains including bulk, solution, and emulsion polymerization. The process involves three key steps: initiation, propagation, and termination. As is shown in Fig. 4, a thermally labile initiator (*e.g.*, azobisisobutyronitrile, AIBN) decomposes under heat (60–100 °C) or light to produce free radicals, which attack monomer double bonds (*e.g.*, vinyl groups in acrylates or styrene), initiating chain growth. Propagation continues *via* successive monomer additions until termination occurs through radical coupling or disproportionation.^53^

Fowler *et al.*^54^ reported the preparation of CO_2_ switchable polystyrene and poly methyl methacrylate (PMMA) latexes by FRP using the cationic switchable surfactants, showing the fact that the aggregation behavior of those latexes largely depended on the amount of initiator, surfactant and polymer concentration in the system. Mihara and coworkers reported the example of redispersible CO_2_ switchable latexes by indicating that addition and removal of CO_2_ led to redispersion and coagulation of the latexes.^55^ The imidazole-functionalized initiator on each polymer chain underwent CO_2_-mediated protonation, thereby enhancing colloidal stabilization of the latex particles. This behavior underscores the potential of CO_2_ responsive moieties in designing switchable surfactants for emulsion-based applications.^12^ The CO_2_ switchable amidine-functionalized latex developed by Zhu *et al.* demonstrated a sustainable pathway for emulsion recycling in coating industries, eliminating the need for traditional chemical stabilizers.^56^ They then designed a reactive surfactant that can dynamically control latex morphology, in line with the principles of circular economy and further advancing green polymerization technology. Currently, the primary challenges associated with FRP include the complexity of the process due to the stringent deoxygenation requirements during synthesis and the broad molecular weight distribution of the resulting products.^57,58^ Nevertheless, owing to its cost-effectiveness, FRP remains an indispensable method for industrial-scale polymer synthesis. Emerging trends emphasize the synergistic integration of FRP with other polymerization techniques to develop more efficient stimulus-responsive synthesis methods.^59^

#### 2.2.2 Reversible addition–fragmentation chain transfer

Reversible addition–fragmentation chain transfer (RAFT) polymerization is a controlled radical polymerization (CRP) technique that enables precise synthesis of polymers with tailored structures, narrow molecular weight distributions, and functional end-groups. RAFT polymerization mechanism shares similarities with conventional FRP in its initiation step, where a radical initiator generates primary radicals to attack monomer units. In the subsequent pre-equilibrium phase, the RAFT agent undergoes reversible chain transfer with propagating radicals, forming a macro-RAFT intermediate. RAFT intermediates regulate chain growth and dispersity through the dynamic exchange between active and dormant chains. Propagation and termination in RAFT polymerization proceed similarly to FRP, although termination is significantly suppressed due to the dominance of the RAFT equilibrium.^60^ The very specific details are demonstrated in Fig. 4.

RAFT polymerization has been effectively utilized to synthesize CO_2_ switchable polymers, exemplified by the preparation of dual CO_2_-and temperature-responsive block copolymers such as poly (diethylaminoethyl methacrylate)-*block*-poly (*N*-isopropylacrylamide) (PDEAEMA-*b*-PNIPAM).^61^ RAFT polymerization technology is currently advancing toward the development of multi-responsive systems (*e.g.*, dual pH/CO_2_-responsive polymers) and sustainable practices (*e.g.*, green solvents, enzyme-mediated RAFT)^62^ to mitigate the instability and potential toxicity associated with the process.^63,64^ Simultaneously, its integration with machine learning for predicting polymerization kinetics is expected to further enhance the performance of responsive materials.^65,66^

#### 2.2.3 Atom transfer radical polymerization

Atom transfer radical polymerization (ATRP) operates through a dynamic equilibrium between propagating radicals and dormant species, which governs its controlled polymerization behavior.^67^ In this mechanism, the majority of polymer chains (Pn) exist as dormant species (Pn–X, where X is a halogen atom), which periodically undergo reversible halogen transfer with transition metal catalysts. The oxidized complex acts as a deactivator, recapturing the propagating radical to generate the dormant species and the activator.^67,68^ This redox cycle maintains an ultralow concentration of active radicals, thereby suppressing termination reactions and ensuring narrow molecular weight distributions.^12,69^ The detailed reaction chains are show in Fig. 4.

Huo *et al.*^70^ demonstrated a CO_2_ regulated self-assembly behavior of an amphiphilic terpolymer. The CO_2_ responsive polymer was integrated with other polymer assemblies through ATRP technology to achieve precise control over the assembly structure.^12^ Simultaneously, the CO_2_ responsiveness of the assembly was significantly enhanced. The versatility of CO_2_ responsive polymers, particularly block copolymers synthesized *via* ATRP, stems from three inherent advantages of the ATRP methodology (1) its capacity to design complex morphologies (*e.g.*, micelles, vesicles): with precise stimuli-responsiveness;^60^ (2) compatibility with surface-initiated polymerizations (SI-ATRP) for functional coatings; (3) broad monomer applicability, ranging from hydrophobic styrene to hydrophilic aminoethyl methacrylates.^66,71^ ATRP is favored for its superior scalability and sustainability, however, its industrial application has lagged due to limitations such as high catalyst cost and slow reaction kinetics.

The synthesis of CO_2_ responsive polymers is primarily achieved through FRP,RAFT and ATRP. FRP demonstrates industrial viability due to its cost-effectiveness, though it exhibits broad molecular weight distributions. RAFT enables precise structure control for complex functionalities through tailored block sequences, while ATRP excels in constructing sophisticated topological structures despite requiring metal catalysts. Table 2 systematically compares these methods in terms of molecular weight regulation, monomer compatibility, and petroleum engineering applications, providing critical guidance for selecting appropriate technologies across different operational scenarios.

**Table 2:** Comparison of synthetic methods of CO_2_ responsive polymers

Content	FRP	Synthetic methods	ATRP
		RAFT	
Reaction mechanism	Radical chain reaction; initiation, propagation, and termination *via* free radicals	Chain transfer to RAFT agent controls polymer growth; reversible transfer between active and dormant chains	Reversible redox equilibrium between active radical and dormant species; transition metal catalyst mediates activation/deactivation
Molecular weight control	Poor, broad	Excellent, narrow	Excellent, narrow
Reaction conditions	Mild conditions (ambient to 120 °C); requires minimal oxygen exclusion	Similar to FRP but oxygen exclusion is necessary; moderate temperature (60–90 °C)	Metal catalysts; oxygen-sensitive (degassing required); moderate temperature (60–100 °C)
Monomer compatibility	Wide range of monomers	Compatible with diverse monomers	Works well with vinyl monomers
Cost	Low	Moderate	High, due to expensive catalysts and ligands
Reaction time	Fast	Moderate	Moderate to slow
Advantages	Simple and cost-effective; suitable for large-scale production	Excellent molecular weight control; ability to synthesize complex structure	Precise control over polymer structure; versatile for various polymer structure
Limitation	Poor molecular weight control; limited end-group functionality	RAFT agent residues may affect final properties	High sensitivity to oxygen and requires rigorous deoxygenation
Oilfield applications	Viscosity enhancers, high-temp and salt resistance additives	High-temp fracturing fluids, fluid loss control	Smart gels, Nanocarriers
Ref.	72 and 73	74, 75 and 76	77 and 78

## 3 Application of CO_2_ responsive materials in drilling engineering

Drilling engineering, serving as a critical link across the entire lifecycle of oil and gas resource development, faces numerous challenges that threaten operational efficiency and reservoir integrity. These challenges include formation fluid erosion, wellbore instability, fracturing fluids performance failure and complex oil–water mixture treatment. CO_2_ responsive materials, characterized by their environmentally triggered dynamic response mechanisms, provide innovative solutions across multiple fronts. These materials can significantly enhance cementing strength by forming adaptive, self-healing barriers that respond to CO_2_ exposure, thereby improving long-term wellbore stability. Additionally, they enable intelligent regulation of fracturing fluids by modulating viscosity and fluid behavior in real time under varying CO_2_ concentrations, optimizing fracture propagation and proppant placement. Furthermore, CO_2_ responsive materials facilitate efficient oil–water separation by altering interfacial properties, allowing for rapid phase demulsification and improved treatment of produced fluids. These advancements not only address critical challenges in drilling operations but also contribute to sustainable and cost-effective oilfield development.

### 3.1 Cementing

**Fig. 5:**
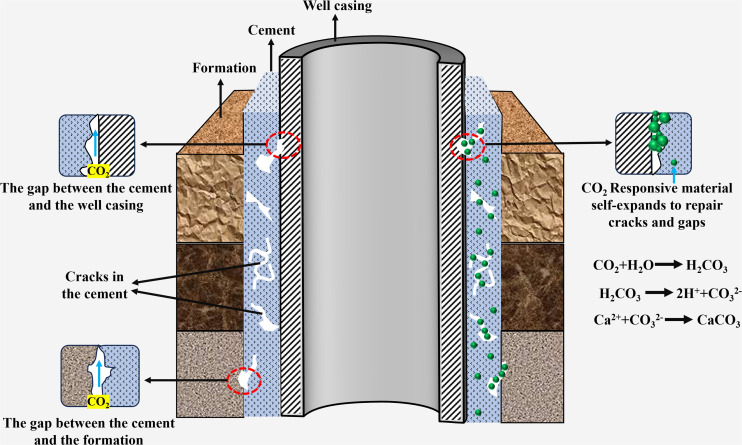
Application of CO_2_ responsive materials in cementing operations.

Cementing refers to the process of injecting cement slurry into the wellbore and casing annulus to form a sealing layer, thereby supporting the wellbore structure to prevent collapse and protecting the casing from corrosion. It is a key technology to ensure drilling safety, optimize production capacity, and maintain long-term stable oil and gas production. In cementing operation, CO_2_ responsive materials facilitate rapid densification and performance optimization of the cement matrix through controlled carbonation reactions. Simultaneously, their responsive properties enable the self-repair of microcracks, thereby enhancing the acid corrosion resistance of conventional cements and extending the wellbore service life (Fig. 5).

In an earlier study, cement crack samples were exposed to CO_2_ saturated water, revealing that CO_2_ reacted with Ca^2+^ in the cement matrix to form calcium carbonate, which filled the cracks and formed a dense layer, resulting in the recovery of peak strength compared to untreated samples.^79^ Subsequently, a cement system resistant to CO_2_/H_2_S corrosion was developed by compounding mineral binders with ordinary portland cement.^80^ CO_2_ responsive materials have also been applied to wellbore surface coatings, providing adaptive corrosion protection in curing environments and enhancing wellbore corrosion resistance.^81^ J. Zhang *et al.*^82^ significantly improved the corrosion resistance of cement by introducing CO_2_ responsive microspheres, which undergo molecular chain crosslinking and membrane reconfiguration in the acidic environment of CO_2_ to form a dense barrier. This barrier effectively blocks the penetration of corrosive media and inhibits the acid-base reactions of cement hydration products, reducing corrosion rates by 70% compared to conventional cement materials. Microcracks often form in cement during the cementing process and are difficult to repair. To address this, Xie *et al.*^73^ developed a CO_2_ responsive hydrogel by FRP with acrylic acid and diethylaminoethyl methacrylate as monomers. This hydrogel can trigger the antipolyelectrolyte effect upon exposure to CO_2_, enabling it to swell and fill microcracks. Experimental results demonstrated that cement containing 0.3% hydrogel exhibited a 1361% strength growth rate after 56 days of repair. Gong *et al.*^83^ added graphene oxide (GO) to cement slurry, triggered the “carbon dot effect” in supercritical CO_2_ (ScCO_2_) environment, induced the hydration product Ca(OH)_2_ to rapidly carbonize into CaCO_3_, and formed a high-polymerization C–S–H gel. Ultimately, the porosity of cement was reduced by 43%, the compressive strength growth rate increased by 14%, and the microstructure of cement was significantly optimized, improving its impermeability and durability in cementing operations.

### 3.2 Fracture

Fracturing fluid is the key medium for fracture and sand-carrying in hydraulic fracturing. Its core function is to form a diversion fracture network and support reservoir transformation through high-pressure injection. The current main fracturing fluids for low-permeability reservoirs include natural guar or slick water with low concentrations of polyacrylamide.^84^ However, conventional fracturing fluids in low permeability reservoirs encounter several challenges, including polymer residue impairing flow conductivity, environmental risks of chemical breakers and instability at high temperature. CO_2_ responsive viscoelastic fracturing fluids overcome these limitations through CO_2_-triggered self-assembly of surfactants, forming worm-like micelles and enabling intelligent switching, thereby avoiding the irreversible degradation associated with conventional fluids.^23^

**Fig. 6:**
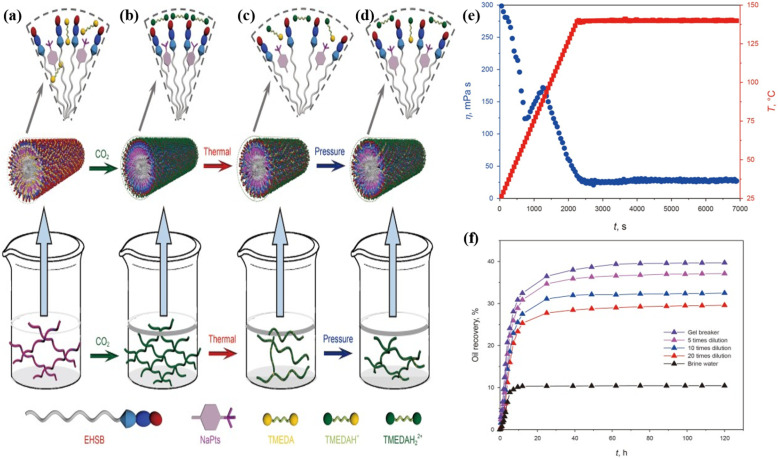
Schematic illustration of self-assembly mechanism of the smart fluid induced by CO_2_, thermal and pressure. (a) Elastic gel, initial state without CO_2_ at 25 °C and atmosphere; (b) stronger elastic gel, after CO_2_-response at 25 °C and atmosphere; (c) worm-like micelle, after CO_2_-response at 80 °C and atmosphere; (d) stronger worm-like micelle, after CO_2_-response at 80 °C and 3.0 MPa; (e) apparent viscosity (170 s^−1^) and temperature as a function of time for the smart fluids with CO_2_ at 3.5 MPa; (f) oil recovery of spontaneous imbibition experiments for gel breaking fluids and brine water at 80 °C. This figure has been reproduced from ref. 87 permission from Elsevier, copyright (2023).

CO_2_ responsive fracturing technology continues to enhance the system performance through the integration of molecular structure design and environmental triggering mechanisms. Early studies focused on the anionic surfactant system, which constructs a dynamic biomimetic baryonic structure *via* CO_2_ protonation, forming a worm-like micellar network, that achieves a viscosity of 25 mPas and a gel-breakage fluid viscosity of 3.2 mPas at 70 °C. However, this system exhibited limited adaptability to high temperatures.^85^ Sun *et al.*^86^ improved the temperature resistance of CO_2_ responsive fracturing fluid to 120 °C (26.2 mPas) by incorporating amphoteric betaine with amine-based surfactants. Moreover, M. W. Gao *et al.*^87^ developed an innovative composite system introducing a temperature-pressure-CO_2_ triple response mechanism. The self-assembled structure changes dynamically with the environment. It is an elastic gel at room temperature and pressure (Fig. 6a). After the injection of CO_2_, the protonation is enhanced to form longer and harder worm-like micelles (Fig. 6b). As the temperature rises, the degree of protonation decreases and the micelles shorten (Fig. 6c). The increase in CO_2_ partial pressure causes CO_2_ to continue to dissolve and the viscosity to further increase (Fig. 6d). Experiments have shown that the system still maintains an effective viscosity of 30 mPas at 140 °C (Fig. 6e). Moreover, the gel-breaking fluid achieves a remarkable oil displacement efficiency of approximately 40% through spontaneous imbibition mechanisms (Fig. 6f), thus advancing development of synergistic fracturing-oil repulsion technology.

**Fig. 7:**
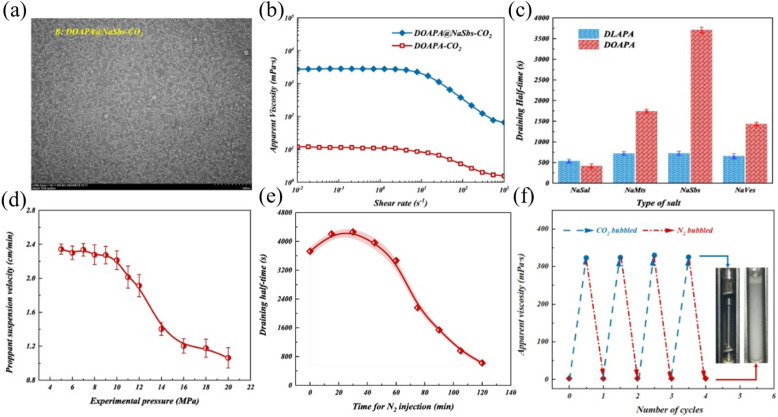
(a) Self-assembled structures of surfactant aggregates in CO_2_ responsive DOAPA-NaSDS systems; (b) shear-dependent viscosity characteristics of foam formulations under varying flow conditions; (c) salt-induced modifications in foam drainage kinetics; (d) pressure-responsive proppant transport performance of CO_2_-activated viscoelastic foams; (e) thermal and pressure stability evaluation of nitrogen-containing foams under reservoir conditions (60 °C, 8 MPa); (f) reversible viscosity modulation through gas switching (CO_2_/N_2_) in smart surfactant systems. This figure has been reproduced from ref. 89 permission from Elsevier, copyright (2025).

Current research focuses on achieving precise control of fracturing fluid viscosity through CO_2_ response mechanisms; however, its sand-carrying capacity under extreme conditions requires further optimization. Surfactant molecules can be induced to self-assemble into worm-like micelles under CO_2_ stimulation, where the entanglement of micelles forms a transient three-dimensional network that significantly enhances viscosity. Simultaneously, the incorporation of polymers further improves fluid stability and optimizes sand-carrying capacity. Samuel *et al.*^88^ developed the S-Gel 38 system, which can maintain a viscosity of more than 100 cP and achieve a proppant suspension time of more than 1 hour at a high temperature of 135 °C by introducing S-Gel 38 polymer. Its 15 gpt system can maintain a viscosity of 70 cP within two hour. The system significantly improves the permeability recovery effect through a controllable gel-breaking mechanism. Field applications have shown that this technology has successfully reduced the amount of acid fracturing fluid used by 50%, while the surface tension of the gel-breaking fluid is stably controlled at 28 mN m^−1^, showing excellent engineering applicability. Traditional foam fracturing fluids generally have problems such as weak suspended sand-carrying capacity, short foam half-life, and residual gel damage to the reservoir. To address the above problems, Zheng *et al.*^89^ developed a recyclable CO_2_ responsive VES-CO_2_ foam fracturing fluid system in a supercritical CO_2_ environment based on CO_2_ responsive surfactants oleamido propyl dimethylamine (DOAPA) and sodium benzenesulfonate (NaSDS). The study showed that under CO_2_ stimulation, DOAPA and NaSDS synergistically formed a worm-like micelle network (Fig. 7a), which increased the zero shear viscosity of the foaming fluid from 12 mPas to 2869.69 mPas (Fig. 7b). It also extends the foam drainage half-life to 3720 s, which was significantly better than the traditional system (Fig. 7c). After N_2_ is injected, it can quickly replace CO_2_ to achieve rapid gel breaking. The system viscosity can be reversibly switched between 2869 mPas (CO_2_ stimulation) and 2.2 mPas (gel breaking). After 4 cycles, the performance remained stable, and the core damage rate was only 8.08% (Fig. 7d–f). However, the foam stability of this system in high-temperature reservoirs still faces challenges, and the subsequent focus needs to be on optimizing its temperature adaptability to expand its engineering adaptability under extreme conditions.

### 3.3 Oil–water separation

A large amount of oil–water mixtures produced by oil development and production contain oils, heavy metals and toxic organic matter, with complex components and difficult to handle.^90^ Although traditional physical and biological combined processes can be handled in stages, they still face problems such as the stability of the emulsion system and the residue of chemical demulsifiers. Traditional oil–water separation materials rely on static wettability changes and cannot effectively handle complex emulsion systems. In contrast, CO_2_ is easy to remove as a gas and has no residue, which meets the needs of green separation, and it can be reversibly acid-base responsive when dissolved in water.^91^ CO_2_ responsive materials can undergo reversible changes in wettability or charge state upon exposure to CO_2_, enabling intelligent control of separation behavior.

**Fig. 8:**
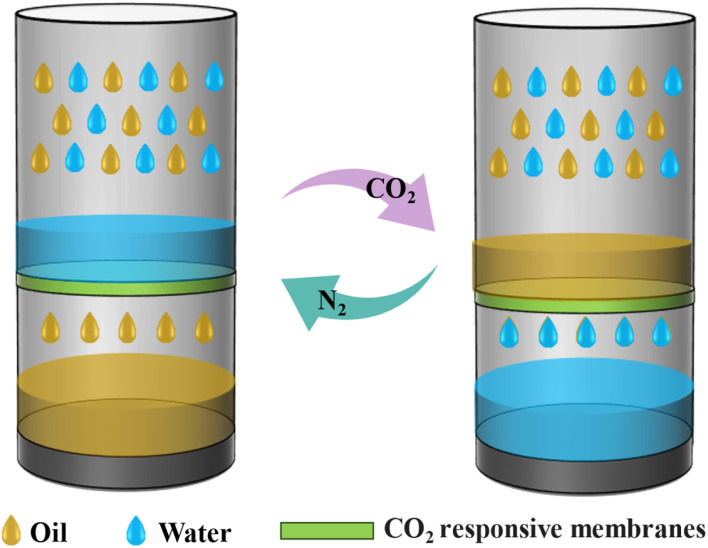
Schematic diagram of oil–water separation regulated by CO_2_ responsive membrane.

CO_2_ responsive membrane materials achieve self-cleaning and oil phase desorption by CO_2_-triggered dynamic reversal of surface wettability. When CO_2_ is injected, a chemical reaction occurs on the membrane surface, altering its wettability from lipophilic to hydrophilic and enabling efficient selective passage of the aqueous phase. Upon switching to N_2_ injection, CO_2_ is physically expelled, causing the membrane surface to revert to a hydrophobic state and preferentially permeate the oil phase (Fig. 8). Qi *et al.*^92^ developed a CO_2_ responsive nanofiber membrane polyacrylonitrile-*co*-poly(diethylaminoethyl methacrylate (PAN-*co*-PDEAEMA)) based on electrostatic spinning technology. Under normal conditions, the tertiary amine groups in the DEAEMA chain segments on the membrane surface were not protonated and showed hydrophobicity. However, upon prolonged exposure to CO_2_, the tertiary amine groups on the membrane undergo protonation, transforming the membrane into a superhydrophilic state. This switchability of the nanofibrous membrane stems from the interaction of CO_2_-induced protonation and hierarchical nanostructures: the PDEAEMA chains extend and increase the surface roughness Ra from 2.99 to 6.48 nm, enabling water permeation while retaining oil due to the hydrophilic/oleophobic properties. Crucially, deprotonation restored the original hydrophobic state of the membrane after 30 min of N_2_ treatment, completely reversing the separation process and confirming the membrane's reversible O/W switching ability. Inspired by the capillary force in nature, Y. Wang *et al.*^93^ fabricated CO_2_-responsive membranes by the capillary force self-assembly (CFCS) method, which utilizes CO_2_ to trigger the hydrophilic–hydrophobic switching property of polymers. The scalable preparation mechanism relies on manipulating capillary force to drive the homogeneous adhesion of poly(diethylaminoethyl methacrylate-*co*-methyl methacrylate (PMMA-co-PDEAEMA) copolymers onto polyester fabric within a 150 μm gap, enabling large-area membrane production up to 3600 cm^2^. This process ensures uniform distribution of CO_2_ responsive tertiary amine groups, as validated by SEM showing consistent surface roughness and EDX mapping confirming homogeneous N element distribution. The membranes achieved more than 99.3% separation of simulated multiphase emulsions such as *n*-butane, silicone oil, and toluene. Moreover, they demonstrated excellent self-cleaning efficiency of up to 99.5% for all emulsion systems through CO_2_/N_2_ switching and maintained stable performance after 20 reuse cycles, offering a novel approach for the large-scale production of stimuli-responsive membranes.

Conventional CO_2_ responsive membrane materials often face the challenges such as slow deprotonation and high energy consumption when processing complex double emulsions (O/W/O or W/O/W), making it difficult to achieve efficient separation. To address these limitations, H. Liu *et al.*^94^ used a two-step coating method to prepare fibers with a primary photothermal responsive coating and a secondary CO_2_ responsive coating, and converted the fibers into dual CO_2_/photothermal responsive films by industrial means. CO_2_ stimulation protonates polymethyl methacrylate (PMMA)-*co*-poly (2-(diethylamino)) ethyl methacrylate (PDEAEMA), rendering the membrane surface superhydrophilic and allowing the permeation of the aqueous phase. Moreover, near-infrared (NIR) light was able to trigger the photothermal effect of graphene oxide (GO), locally heating the membrane to 140 °C and inducing rapid deprotonation of PDEAEMA to restore lipophilicity within 1 min, allowing the oil phase to pass through efficiently. Compared to traditional CO_2_ membranes relying on nitrogen purge or overall heating, this material reduces the deprotonation time by 95% and achieves precise separation of double emulsions with an efficiency exceeding 99.6%. Similarly, D. Yan *et al.*^95^ achieved continuous separation of complex ternary mixtures of heavy oil, water, and light oil by leveraging the protonation–deprotonation transition of PDEAEMA, providing valuable insights for the development of novel membranes with switchable wettability. Despite these advancements, the continuous separation of multicomponent mixtures remains a significant challenge, necessitating breakthroughs in the integration of multiple response mechanisms.

In summary, CO_2_ responsive materials achieve environmentally adaptive functionality through precise molecular design, enabling intelligent responsiveness to CO_2_ stimuli. These materials have been widely applied in various aspects of oil and gas operations, including cementing, fracturing, and oil–water separation. In cementing, they enhance wellbore integrity by forming self-healing barriers in response to CO_2_ exposure. In fracturing, they enable real-time viscosity regulation, improving fluid efficiency and fracture control. Additionally, in oil–water separation, they facilitate efficient phase demulsification, optimizing produced fluid treatment. The main chemical compositions of these materials, along with their key application are detailed in Table 3.

**Table 3:** Application of CO_2_ response materials in drilling engineering

Application	Key chemicals	Temperature tolerance	Beneficial effects	Ref.
Cement slurry	Self-synthesized new materials environment responsive microsphere (ERPM)	—	Corrosion depth reduced by 70% and compressive strength reduction by <12%	82
	Polypropylene calcium salt-dimethylaminoethyl methacrylate hydrogel (Ca-PAD)	—	The self-repair strength reached 1361% in 56 days, and the volume repair rate increased to 61.7% in 14 days	73
	Graphene oxide (GO)	—	The compressive strength growth rate reaches 2.9 Mpa per day, the porosity decreases by 43%	83
Fracturing fluid	Sodium dodecyl sulfate (SDS), 2,6,10-trimethyl-2,6,10-triazaundecane (TMTAD)	70 °C	The viscosity at 70 °C is 25 mPas, the viscosity after breaking is 3.2 mPas, and the clay anti-swelling rate is 91.3%	85
	Erucic acid amide hydroxypropyl sulfobetaine (EAHSB), erucic acid amide propyl dimethylamine (EKO)	120 °C	The viscosity is 26.2 mPas at 120 °C, and the core damage rate is only 7.48%	86
	*N*-Erucylamidopropyl-*N*,*N*-dimethyl-3-ammonio-2-hydroxy-1-propane-sulfonate (EHSB), *N*,*N*,*N*′,*N*′-tetramethyl-1,3-propanediamine (TMEDA)	140 °C	The viscosity at 140 °C is 30 mPas, the permeability damage rate is 3.33%, and the oil displacement efficiency of the gel breaking fluid is 40%	87
	A cationic micropolymer S-gel 38	135 °C	Maintains viscosity above 100 mPas at 135 °C, proppant suspension time over 1 hour	88
	Oleylamide propyl dimethylamine (DOAPA), sodium benzenesulfonate (NaSbS)	60 °C	The proppant settling velocities in CO_2_ were 2.34 cm min^−1^, the fracturing fluid performance did not change after 4 cycles of CO_2_/N_2_	89
Oil–water separation membrane	Poly(diethylaminoethyl methacrylate) (PDEAEMA)	—	Separation efficiency > 99%, performance is stable in pH = 2–12, and salt resistance reaches 10%	92
	Poly(diethylaminoethyl methacrylate-*co*-methyl methacrylate) (PMMA-co-PDEAEMA)	—	The CFCS method is used for synthesis, which is conducive to large-scale production, and the self-cleaning rate >99.5%	93
	Graphene oxide (GO), poly(diethylaminoethyl methacrylate) (PDEAEMA)	—	Effectively separate double emulsions, separation efficiency > 99.6%	94
	Poly(vinyltrimethoxysilane)-*co*-poly(*N*,*N*-dimethylaminoethyl methacrylate) (PVTMS-*co*-PDMAEMA)	—	The continuous separation efficiency of heavy oil–water-light oil mixture reached 99.9%, exhibits stable performance after repeated use	95

## 4 Application of CO_2_ responsive materials in reservoir engineering

The primary challenge in reservoir engineering is to precisely regulate the complex subsurface seepage flow to achieve efficient and sustainable resource exploitation. However, the inherent heterogeneity of reservoirs, the difficulty in controlling gas flow, and the persistent risk of sealing leakage have long constrained the effectiveness and applicability of conventional materials. These limitations not only reduce recovery efficiency but also increase operational risks and costs. In this context, CO_2_ responsive materials, with their environmentally triggered adaptive properties, offer a transformative solution. By dynamically responding to changes in reservoir conditions, these materials can effectively seal gas channeling, minimize leakage risks, and enhance oil recovery. Moreover, they play a pivotal role in advancing CCUS technologies, which have gained increasing prominence in recent years as a critical strategy for mitigating carbon emissions. Through their intelligent adaptability and multifunctional capabilities, CO_2_ responsive materials can help increase oil and gas production while reducing the negative environmental impacts of hydrocarbon extraction.

### 4.1 Gas channeling plugging

**Fig. 9:**
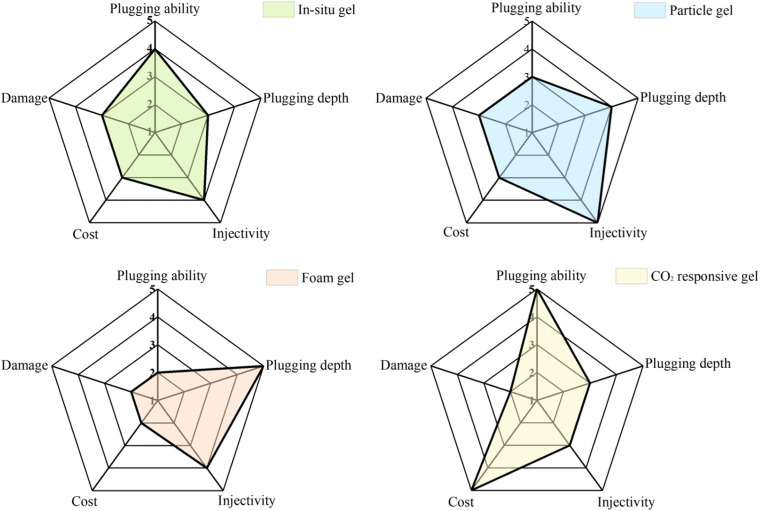
Comparison of performance of different gel materials in plugging gas channel.

As global demand for crude oil continues to increase, the exploitation of low- permeability hydrocarbon resources has become increasingly crucial.^96^ Although CO_2_ flooding technology can significantly enhance oil recovery by reducing viscosity and increasingly solubility, it tends to preferentially flow through high-permeability channels in heterogeneous reservoirs, leading to instability and reduced efficiency at the displacement front.^97^ Technologies such as water-alternating-gas (WAG) injection and foam/gel sealing have been employed to regulate CO_2_ flow. However, traditional gel materials often exhibit poor injectability and insufficient long-term blocking performance under extreme conditions. In recent years, intelligent responsive materials, such as CO_2_ responsive gels and foams, have been continuously optimized to achieve dynamic and adaptive gas plugging by sensing the reservoir's CO_2_ environment, thereby triggering phase transitions and enhancing performance.^98–101^Fig. 9 compares the performance of conventional gels and CO_2_ responsive gels in plugging gas channeling, focusing on plugging capacity, injectability and cost-effectiveness. Particle gel has good injectability due to its small particle size and good fluidity, but its plugging ability is weak. Foam gel has less damage to the reservoir, but it also has the problem of insufficient plugging ability. CO_2_ responsive gel has strong plugging ability and low damage to the reservoir due to its intelligent response characteristics. However, its production process still needs to be further optimized to reduce the cost.

To address the gas channeling problem caused by fractures during CO_2_ flooding in ultra-low-permeability reservoirs, Du *et al.*^102^ reported a coupled system of CO_2_ responsive gel particles (CRPGP) and worm-like micelles (CTWM). After exposure to CO_2_, CTWM transformed from a spherical to a worm-like structure and formed a dense network with CRPGP through hydrophobic interactions, which increased the viscosity by 225 times and the plugging efficiency by 99.2%. In order to solve the challenge of poor injectability during the gel plugging process, Gu *et al.*^103^ synthesized a CO_2_ responsive microgel based on chitosan. After exposure to CO_2_, the gel particle size of this material can shrink rapidly, significantly improving the injection performance, and its flow properties can be adjusted by injecting N_2_. Based on the traditional gel swelling-bridging plugging mechanism, M. L. Shao& Liu^104^ developed a core–shell structured CO_2_ responsive nanoparticle blocking agent, whose particle size can expand from 96 nm to 221 nm, effectively plugging high permeability channels. The rigid styrene component in the plugging agent limits its excessive expansion, thereby preventing plugging failure caused by shear.

**Fig. 10:**
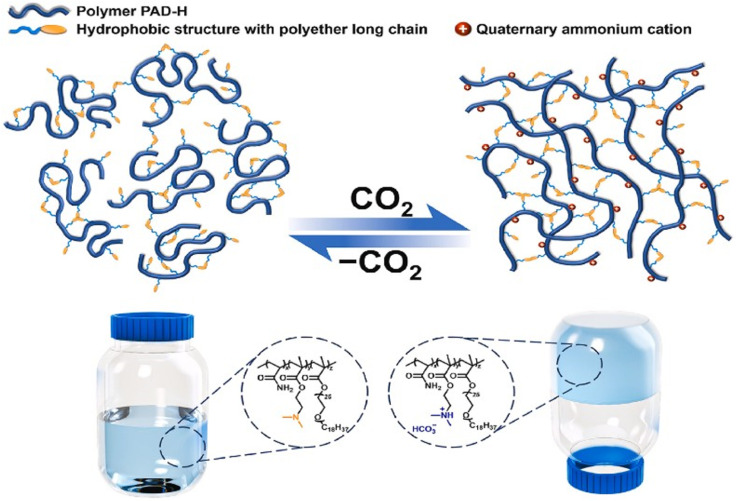
Schematic diagram of CO_2_ responsive viscosity-increasing mechanism of PAD-H. This figure has been reproduced from ref. 106 permission from Elsevier, copyright (2024).

Traditional foams have also been reported to be used to plug gas channeling due to their good injectability, but there are challenges such as poor foam stability and low plugging strength. Q. Gao *et al.*^105^ synthesized a CO_2_ responsive foam (CRF) using sodium lauryl ether sulfate (LES) and diethylenetriamine (DETA), which modulates the solution viscosity through CO_2_/N_2_ stimulation, achieving a balance between low injection pressure and high plugging performance. Experimental results demonstrated that the half-life of CRF was 13 times longer than that of conventional CO_2_ foam and exhibited a stronger resistance factor in high-permeability cores. X. Huang *et al.*^106^ designed a CO_2_ responsive polymer PAD-H by introducing a hydrophobic structure containing polyether chains. The tertiary amine groups in PAD-H are protonated in CO_2_, generating electrostatic repulsion and forming a three-dimensional network structure through hydrophobic association. This network structure significantly enhances the strength of the gel, increasing the plugging success rate to over 95% (Fig. 10). Although foam gels and polymer gels exhibit certain advantages, their effectiveness still requires validation through large-scale field applications.^107^

### 4.2 Enhanced oil recovery

**Fig. 11:**
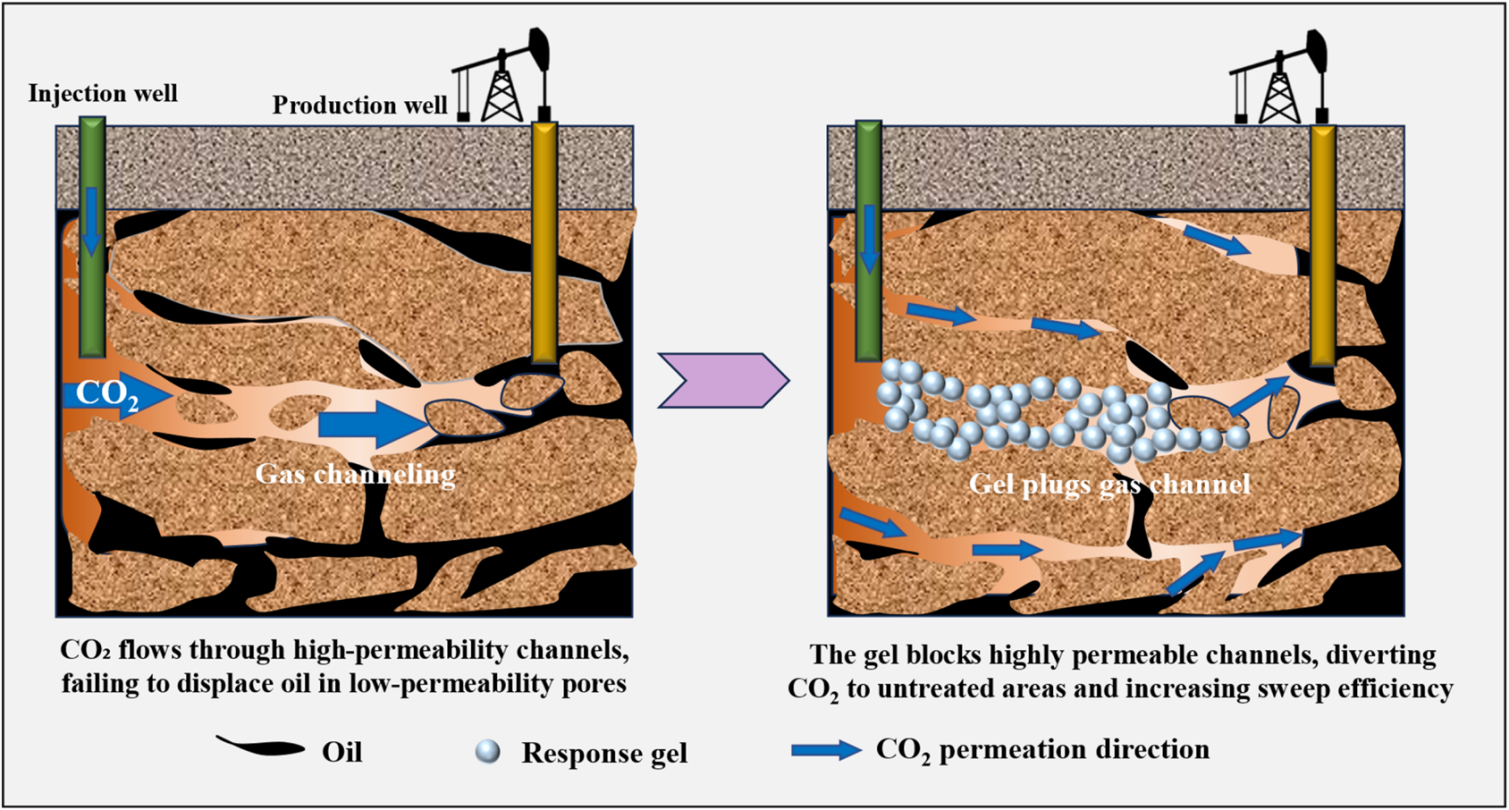
Schematic diagram of CO_2_ responsive gel plugging gas channel for enhanced oil recovery.

CO_2_ responsive materials can dynamically adjust fluid viscosity based on CO_2_ concentration, inhibiting the flow of plugging gels in high-permeability formations and thereby enhancing the sweep efficiency of displacement media.^34,108^ Additionally, CO_2_ responsive materials can also act as surfactants to continuously reduce oil–water interfacial tension, and promote the stripping of residual oil from the formation^109,110^ Fractured reservoirs generally have the problem of low CO_2_ displacement efficiency. CO_2_ responsive gel can achieve the synergistic effect of “plugging high permeability layers and displacing low permeability layers”. During the injection state, low-viscosity fluids enter high-permeability fractured formation, where the smart materials in the fluid respond upon contact with CO_2_, increasing viscosity and gradually exhibiting viscoelastic gel properties. This transformation effectively blocks CO_2_ gas channels in fractures and pore channels. Since the gas channel is sealed, CO_2_ can easily penetrate and diffuse in the low-permeability reservoir, improving the sweep efficiency of CO_2_ and ultimately enhancing the oil and gas recovery (Fig. 11).

**Fig. 12:**
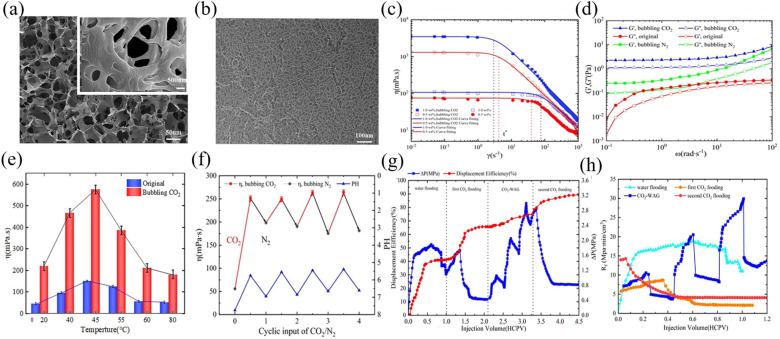
(a) SEM image of hydrogel following CO_2_ induction; (b) TEM image of hydrogel following CO_2_ induction; (c) the viscosity-shear rate relationship of samples at 25 °C with CO_2_ bubbling or non-bubbling. The dotted line represents the position of the critical shear rate; (d) the dynamic moduli of 0.5 wt% samples before and after CO_2_ uptake on the angular frequency at 25 °C; (e) CO_2_ switchable thickening performance during the CO_2_/N_2_ bubbling cycle at 25 °C (concentration: 0.5 wt%); (f) the apparent viscosity of 0.5 wt% sample with CO_2_ and without CO_2_ at different temperatures (g) relationship between oil recovery and Δ*P* with HCPV; (h) relationship between seepage resistance and HCPV under different injection methods. This figure has been reproduced from ref. 112 permission from Elsevier, copyright (2025).

Recent studies have shown that surfactant-based CO_2_ responsive gel systems have shown significant potential in improving oil recovery. A reversible system was constructed based on the long-chain tertiary amine surfactant *N*,*N*-dimethyl erucamide tertiary-amine(DMETA), and the viscosity was reversibly switched through the self-assembly of worm-like micelles (WLMs) triggered by CO_2_, ultimately enhancing the oil recovery rate by 21.7%.^33^ Xin *et al.*^111^ developed a CO_2_ responsive gel system using long-chain alkylamidopropyl dimethyl tertiary amine, which expands the sweep efficiency by uniformly displacing the front edge and reduced the viscosity of crude oil, increasing the oil recovery by 23.92%. Similarly, Fang *et al.*^112^ further designed an irreversible hydrogel based on a long-chain tertiary amine surfactant (HXB-2), which formed a three-dimensional worm-like cross-linked network (Fig. 12a and b) through carboxyl protonation and electrostatic adsorption of bicarbonate. The viscosity of the 0.5wt% solution after CO_2_ triggering reached 1117 mPas (Fig. 12c), and showed elastic response (Fig. 12d). The viscosity could still remained 4 times the initial value at high temperature (Fig. 12e), and the viscosity was only partially restored after N_2_ treatment (Fig. 12f), proving its irreversibility. Core experiments show that during alternating water and gas injection, the displacement pressure increased from 0.416 MPa to 2.423 MPa, the maximum seepage resistance reached 29.45 MPa min cm^−3^, and enhance oil recovery by 24.6% (Fig. 12g and h). After secondary CO_2_ flooding, the oil recovery increased to 89.23%, and the plugging rate reached 94.1%. Surfactant-based CO_2_ responsive materials have the advantages of being mild, safe and economical. In the future, their large-scale production and on-site application can be promoted by further optimizing the synthesis process.

The previous paragraph has introduced the application of CO_2_ responsive materials in chemical flooding such as surfactant flooding and polymer flooding to enhance oil recovery (EOR). In fact, common EOR methods also include thermal flooding, other gas flooding and so on. In recent years, these technologies have shown remarkable results in improving oil recovery through innovative combination with CO_2_ responsive materials.^113^ Tian *et al.*^114^ used acrylamide (AAm), *N*,*N*-dimethylaminoethyl acrylamide (DMAEMA) and [2-(methacryloyloxy)ethyl]dimethyl-(3-sulfopropyl)ammonium hydroxide (SBMA) as raw materials, thermal and CO_2_ dual-responsive smart polymer microgels (SPMs) were synthesized by solution copolymerization and cross-linking technology. When the temperature is higher than 65 °C, the SBMA unit swells due to the thermal induction of the SBMA unit to destroy the intramolecular electrostatic effect; when encountering CO_2_, the tertiary amine group of the DMAEMA unit is protonated to produce electrostatic repulsion, causing the microgel to swell secondary. This technology has firstly promoted the application of CO_2_ responsive materials in thermal flooding. In the future, in scenarios such as steam flooding, the synergistic effect of temperature sensitivity and CO_2_ response can be further used to complete the plugging of high-permeability channels and dynamic profile adjustment, thereby improving the recovery rate.^115^ Additionally, gases like N_2_ and CH_4_ can potentially combine with CO_2_ responsive materials to enhance oil recovery. For instance, N_2_ can be integrated with CO_2_ responsive foams to regulate foam stability *via* gas switching in water-alternating-gas injection, enabling plugging of high-permeability channels. In CH_4_ miscible flooding, CO_2_ responsive materials can adjust their swelling degree according to gas composition changes, optimizing fluid mobility control.^116,117^ Overall, CO_2_ responsive materials show enormous potential in EOR. Future research should focus on integrating multiple EOR methods.

### 4.3 CO_2_ geological storage

**Fig. 13:**
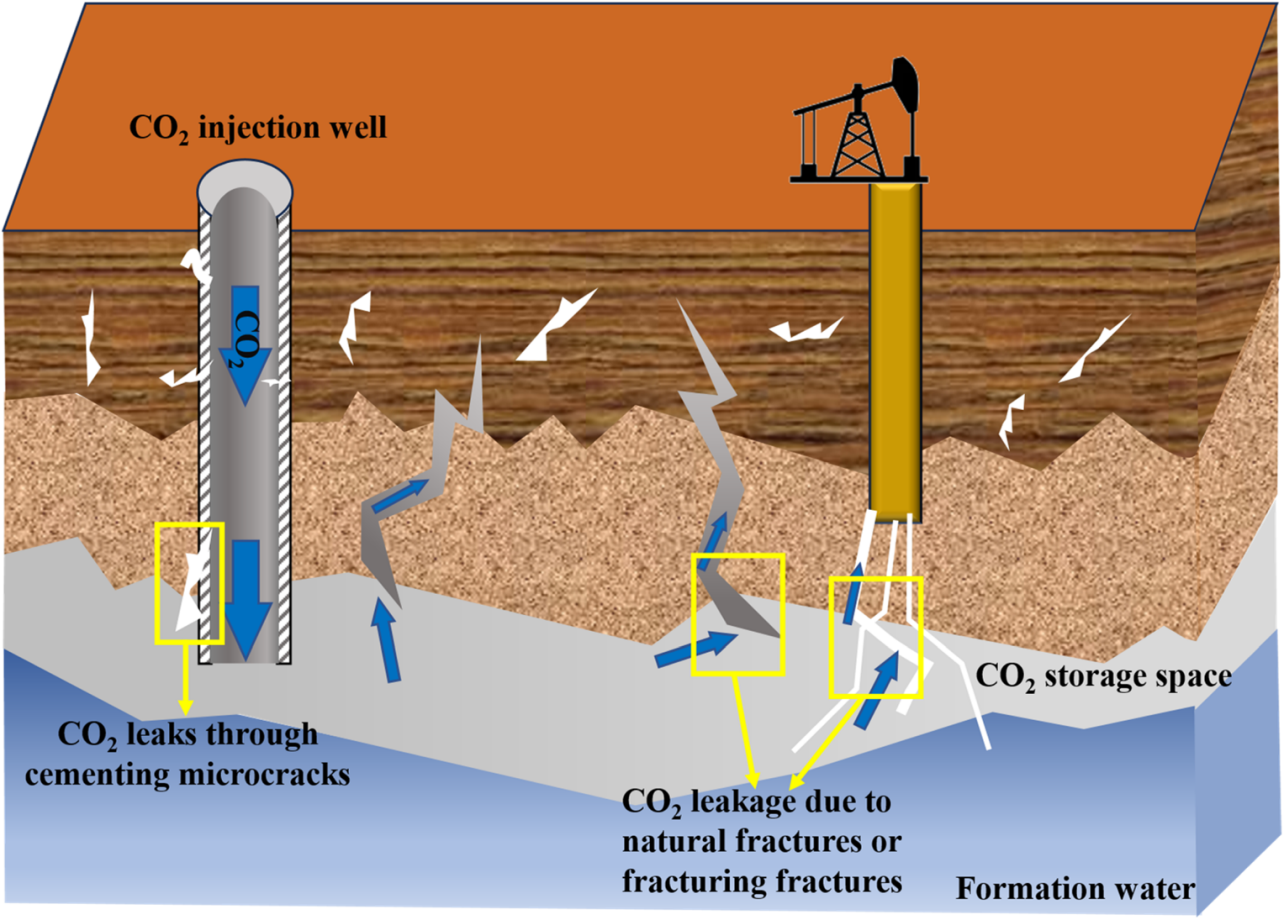
Schematic diagram of CO_2_ leakage during geological burial of CO_2_.

Greenhouse gas emissions from industrial activities, including oil and gas exploration and development, have intensified global warming and triggered ecological disasters such as glacier melting and sea level rise, prompting countries to accelerate their pursuit of carbon neutrality. As a key technology to achieve this goal, carbon sequestration can achieve long-term storage by injecting CO_2_ into deep underground rock formations, including abandoned oil and gas reservoirs, saline layers, and coal seam.^10,118^ However, natural cracks in the formation or defects in the wellbore may cause CO_2_ leakage, posing environmental and safety risks (Fig. 13). Although traditional leak prevention methods such as cementing or artificial barriers are widely used, they face problems which involve material corrosion and delayed repair.^31,119^ CO_2_ responsive materials are born out of demand. When CO_2_ leakage alters local pH or ion concentrations, CO_2_ responsive materials can rapidly trigger swelling, solidification or mineralization reactions, autonomously forming a dense barrier within the leakage pathway. Additionally, it can synergistically grow with the surrounding rock formations, providing a smarter and more reliable technical means for carbon sequestration.^120^

Currently, the CO_2_ responsive materials sealing mechanism for porous or fractured media primarily involves two processes: (1) the gelation reaction, which forms a physical barrier to block fluid flow; (2) the introduction of a CO_2_ sensitive particle suspension system that undergoes a phase transition upon CO_2_ exposure, gradually forming a dense stacking structure that seals the pore network.^121^ H. Wu *et al.*^122^ achieved dynamic sealing by combining acrylamide (AM) and *N*-(3-(dimethylamino)propyl) methylacrylamide (DMAPMA) as monomers and polyethylene amide (PEI) as a cross-linking agent to form a three-dimensional porous network. CO_2_ triggers the protonation of tertiary amines, inducing a sol–gel phase transition, which increases the subsequent water injection plugging efficiency to 96.2%. Methyl methacrylate-based gel is another typical CO_2_ responsive plugging material. It can expand 20 times in volume at low pH values and maintain structural stability, especially at high temperature and high pressure.^123^ In addition, the CO_2_ responsive polymer microspheres can dissolve or cross-link when exposed to CO_2_, enhancing the plugging ability through a dual mechanism of physical plugging and chemical bonding.^124^ Y. Zhao. *et al.*^110^ used a two-step method to synthesize a CO_2_ responsive dual-network gel system, in which 2-acrylamido-2-methylpropane sulfonic acid (AMPS) formed the first layer of rigid inner network, and acrylamide (AM) and polyethyleneimine (PEI) synthesized the second layer of flexible outer network. This dual-network structure significantly improved the mechanical ability and resistance to CO_2_ flushing of the gel, and improved the problems of poor injectability and plugging performance of traditional gels, thereby improving the success rate of carbon sequestration. S. Chen *et al.*^125^ experimentally compared the sealing performance of three individual systems (polymer gel, CO_2_ responsive foam, and CO_2_ responsive thickened polymer), as well as their combinations. It was found that the polymer gel demonstrated the highest plugging efficiency with 86.13%, but its injection performance was relatively poor. In contrast, the CO_2_ responsive thickened polymer exhibited the lowest plugging performance at 23.7%, but when combined with the foam, this hybrid system could plug CO_2_ gas channeling with an efficiency of 95%. Currently, CO_2_ responsive sealing materials require further improvements in acid resistance, long-term stability and environmental friendliness.^126^ Future research should focus on the development of smart gels and the investigation of synergistic effects of relevant additives on gel performance and plugging efficiency, with the goal of enhancing the mechanical strength and corrosion resistance of the gel, thereby broadening its range of applications.

In summary, CO_2_ responsive materials can intelligently adjust fluid viscosity, wettability, and plugging strength based on CO_2_ concentration in the reservoir. These materials have been widely applied in gas channeling plugging, enhance oil recovery, and CCUS, providing precise, long-lasting, and stable solutions for the development of complex oil and gas reservoirs and carbon sequestration. The specific response mechanisms and main results of CO_2_ responsive materials in reservoir engineering applications are summarized in Table 4.

**Table 4:** Application of CO_2_ response materials in reservoir engineering

Application	Key chemicals	Mechanism	Beneficial effects	Ref.
Plug gas channeling	CO_2_ responsive gel particles (CRPGP),CO_2_ responsive wormlike micelles (CTWM)	CO_2_ triggers the hydrophobicity of the micellar gel system	The viscosity of the micelle system increased by 225 times, and the plugging efficiency reached 99.2%	102
	Chitosan (CS), *N*-(3-(dimethylamino)propyl) methylacrylamide (DMAPMA)	CO_2_-induced protonation of chitosan amino groups and reconstruction of the hydrophobic network	The sol/gel state of the system can be reversibly switched by injecting N_2_/CO_2_	103
	Styrene (st), dimethylaminoethyl methacrylate (DMAEMA), acrylamide (AM)	Nanoparticles enhance plugging strength and stability	The system performance can remain stable under high temperature and high salt conditions	104
	Lauryl ether sulfate sodium (LES), diethylenetriamine (DETA)	CO_2_ triggers the formation of worm-like micelles and dynamically regulates viscosity to block gas channeling	CO_2_ responsive foam exhibits a half-life 13 times longer than that of conventional foams	105
	*N*-(2-(methylpropenoxy) ethyl)-*N*,*N*-dimethyloctadecane ammonium bromide(*H*_b_)	CO_2_-triggered protonation of tertiary amines synergizes viscosity enhancement	The system viscosity is increased by 360 times, and the plugging efficiency is >95%	106
Enhanced oil recovery	*N*,*N*-dimethyl octylamide-propyl tertiary amine (DOAPA),sodium p-toluene sulfonate (SPTS)	CO_2_-triggered protonation reaction caused the spherical micelles to transform into worm-like micelles to form highly viscoelastic gels	Mixing 4.4 wt% DOAPA and 2.0 wt% SPTS can enhance oil recovery by 20%	34
	*N*,*N*-dimethyl erucamide tertiary-amine (DMETA)	CO_2_ induces protonation of the solution to form worm-like micelles and reduce crude oil viscosity and interfacial tension	Viscosity can be switched reversibly, and the oil recovery is enhanced by 21.7%	33
	Long-chain alkyl acid amidopropyl dimethyl tertiary amine	CO_2_ triggers worm micelle network to plug fractures	The oil recovery of low permeability core enhanced from 39.78% to 63.7%	111
	Z-2-(3-(docos-13-amido)propyl) dimethylammonium) propanoate (HXB-2)	CO_2_ induces surfactant protonation to form micelles to improve sweep efficiency	The viscosity increased by 4.53 times after CO_2_ bubbling, and the oil recovery enhanced by 23.53%	112
Carbon sequestration	Acrylamide (AM) and *N*-[3-(dimethylamino) propyl] methacrylamide (DMAPMA)	Protonation for sol–gel phase transition	The gel tensile strength reaches 0.65 N and the bonding force reaches 4264 Pa	122
	Methyl methacrylate gels	Protonation and solvation reactions of gel molecular chains	Stable structure under high temperature and pressure	123
	Crylamido-2-methylpropane sulfonic acid (AMPS), acrylamide (AM),polyethyleneimine (PEI)	CO_2_ responsive expansion to seal cracks	The double network structure improves the gel's resistance to CO_2_ flushing and mechanical strength	110
	Chromium stabilizer, sulfate foaming agent, foam stabilizer	Synergistic blocking of CO_2_ responsive foam, gel and polymer	The CO_2_ responsive thickening polymer and foam synergistic plugging system has the best CO_2_ storage effect	125

## 5 Prospects and challenges

CO_2_ responsive materials have demonstrated significant potential in hydrocarbon extraction due to their environmentally friendly nature and reversible regulation capabilities. Unlike traditional materials that rely on chemical additives, CO_2_ serves as a natural triggering medium, activated by *in situ* concentration or external gas injection, effectively avoiding chemical residue pollution and reducing operational costs. Current advancements have highlighted their application across multiple scenarios in the field of petroleum engineering. For cementing engineering, these materials enhance corrosion resistance and prolong wellbore integrity through dynamic carbonation control of cement matrices. In reservoir stimulation, they optimize proppant transport and placement by intelligently modulating the viscosity of fracturing fluids in response to CO_2_ exposure. Additionally, in produced fluid treatment, they facilitate efficient oil–water separation by altering interfacial wettability, enhancing phase demulsification and improving treatment efficiency.

A particularly promising application in the use of CO_2_ responsive gels, which can precisely plug fractures and pores due to their good injectability and plugging strength. CO_2_ response gels can improve the sweep efficiency of CO_2_ flooding in low-permeability reservoirs by plugging gas channels in high-permeability formations, ultimately achieving the goal of enhancing oil recovery. However, their full potential remains underexplored, particularly in addressing complex reservoir heterogeneities. Moreover, current research predominantly focuses on plugging and displacement functions, with limited exploration of their broader role in carbon capture and utilization. As the global energy industry accelerates its transition under the “Dual Carbon” strategy, the development of next-generation intelligent material systems that seamlessly integrate reservoir adaptability with carbon sequestration capabilities will be essential for achieving both enhanced recovery and sustainable resource management.

**Fig. 14:**
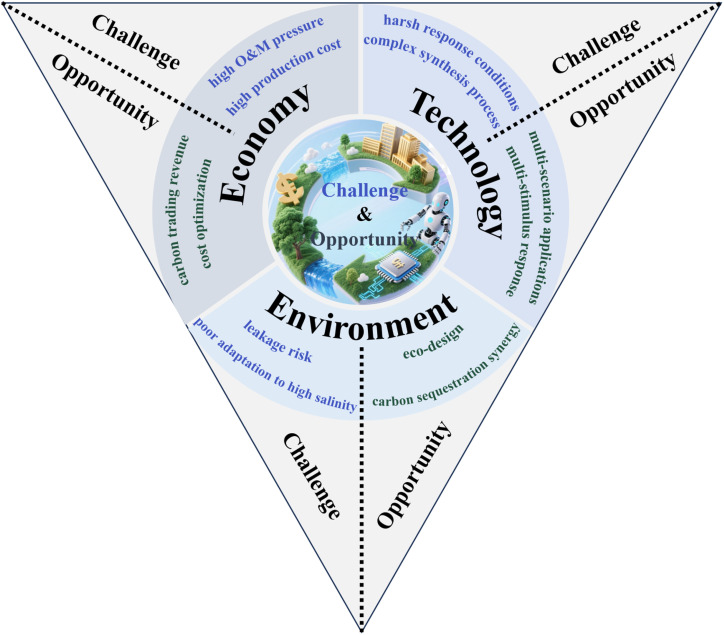
Challenges and opportunities of CO_2_ responsive materials in oilfield extraction: a tripartite analysis of technical, economic, and environmental dimensions.

Despite their promising prospects, the practical application of CO_2_ responsive materials remains to face multiple challenges across three dimensions in oil and gas operations (Fig. 14). Technically, their responsive behavior is constrained by narrow environmental pH ranges, leading to performance degradation in acidic gas reservoirs or alkaline waterflooding zones. Most practiced systems require high CO_2_ concentrations for activation, limiting sensitivity in low-concentration environments like depleted reservoirs. The complex synthesis processes and poor high-salinity tolerance further restrict field applicability. Economically, industrial-scale production incurs high costs (*e.g.*, specialized monomers for guanidine groups), while balancing CO_2_ diffusion efficiency with long-term material stability elevates operational maintenance pressures. Environmentally, potential CO_2_ leakage from inadequate plugging and limited adaptability to high-salinity formations pose ecological challenges.

To address these challenges, future research should focus on three complementary dimensions. In the technical dimension, multi-stimulus systems that combine CO_2_ responsiveness with light, magnetic fields, or temperature to help materials adapt to different reservoir conditions are to be developed. Simultaneously, innovating microgel structures with precise pore and surface designs to improve CO_2_ diffusion and material stability is pressing. In the environmental dimension, using eco-friendly designs to reduce ecological impact and combining carbon sequestration with leakage prevention through CO_2_ mineralization to boost carbon fixation efficiency are necessary. In the economic dimension, generating income from carbon trading *via* CCUS-certified applications is of great importance, thus lowering costs with scalable polymerization techniques to build a sustainable model is also required at the same time. By aligning material innovation with engineering practices, CO_2_ responsive materials can provide sustainable solutions for the green development of complex hydrocarbon reservoirs. These advancements will enable the petroleum industry to dynamically balance the dual objectives of enhancing oil recovery and achieving carbon neutrality, paving the way for a more sustainable and efficient future in hydrocarbon resource utilization.

## 6 Conclusions

This review underscores the promising potential of CO_2_ responsive materials in oil and gas engineering, highlighting key advancements in the past five years. It examines the compatibility of material response mechanisms with reservoir environments, and emphasizing their engineering value under extreme conditions and low-carbon objectives.

(1) CO_2_ responsive materials utilize functional groups such as guanidine, amidine, imidazole, and tertiary amine to undergo reversible protonation reactions with CO_2_, inducing molecular conformational changes that modulate physicochemical properties like viscosity and wettability. Additionally, FRP, featured as low cost, compatibility with large-scale processes but uncontrolled architecture, batch-to-batch variability, and thermal instability; RAFT, featured as its tolerance to functional groups/protic media, ideal for stimuli-responsive polymers but purification challenges due to sulfur residues; ATRP, featured as high-fidelity surface grafting but metal contamination, oxygen sensitivity, and costly catalyst removal enable the responsiveness of polymers precisely tailored, granting these materials environmentally adaptive properties.

(2) CO_2_ responsive materials enable intelligent control of key processes in oil and gas production by triggering phase transitions and structural reorganization upon CO_2_ exposure. They are widely applied in drilling and reservoir engineering, significantly enhancing wellbore stability during drilling operations and improving oil recovery. Adaptive plugging systems increase sweep efficiency and mitigate gas channeling, while dynamic wettability inversion effectively addresses complex emulsification challenges in oil–water separation.

(3) Despite their revolutionary potential, challenges remain in the practical deployment of CO_2_ responsive materials, including stability under extreme conditions, limited response ranges, and issues with scalability and process compatibility. Future research should focus on optimizing synthesis methods for high-temperature, high-pressure-resistant polymers to facilitate the large-scale adoption of CO_2_ responsive materials in intelligent drilling and CCUS.

## Author contributions

Qiang Li: writing (original draft), investigation, visualization; Xuanze Zhu: writing (original draft), investigation, visualization; Jiuyi Chen: writing (original draft); Xionghu Zhao: writing (review& editing), project administration, supervision, validation.

## Conflicts of interest

There is no conflict to declare.

## Data Availability

No primary research results, software or code have been included and no new data were generated or analysed as part of this review.
